# Use of combined transarticular pinning and external skeletal fixation for the reduction and stabilization of multiple metatarsophalangeal luxations in a cat

**DOI:** 10.1177/2055116920904465

**Published:** 2020-02-11

**Authors:** Valentine D Verpaalen, Daniel D Lewis, Erin G Porter

**Affiliations:** Department of Small Animal Clinical Sciences, College of Veterinary Medicine, University of Florida, Gainesville, FL, USA

**Keywords:** Metatarsophalangeal, luxation, transarticular pinning, external skeletal fixation

## Abstract

**Case summary:**

A 1-year-old spayed female domestic shorthair cat presented for evaluation of a non-weight bearing right pelvic limb lameness after falling from a 4 m height. On orthopedic examination there was substantial swelling and pain on manipulation of the right pes. Radiographs were obtained under sedation, and these revealed dorsoproximal luxations of the third, fourth and fifth metatarsophalangeal joints, and lateral rotation of the second digit. Closed manual reduction under sedation was unsuccessful and open reduction under general anesthesia was therefore performed. Combined transarticular pinning and external skeletal fixation were performed to maintain reduction of the third and fourth digits. Marked postoperative swelling of the distal pes and internal rotation of the third and fourth digits were noted within 24 h of surgery. Three weeks postoperatively, the cat had a persistent weight bearing right pelvic limb lameness and minor pin tract inflammation. All implants were removed and the limb was splinted for 1 week. Internal rotation and pin tract inflammation had resolved at the time of splint removal, and the lameness resolved within 6 weeks of surgery. The cat was not lame, but radiographs revealed mild-to-moderate degenerative osteoarthrosis when the cat was evaluated 6 months after surgery.

**Relevance and novel information:**

There are limited reports describing metatarsophalangeal luxations in cats. Although several surgical techniques have been advocated, specific outcomes in clinical cases have not been reported. This report describes the clinical application and outcome of combined transarticular pinning and external skeletal fixation for the management of multiple metatarsophalangeal luxations in a cat.

## Case description

A 1-year-old spayed female domestic shorthair cat weighing 4.3 kg presented for evaluation of an acute (<24 h) non-weight bearing right pelvic limb lameness sustained after falling approximately 4 m in height. The cat was reluctant to ambulate and would not bear weight on the right pelvic limb. There was substantial swelling and pain on manipulation of the right metatarsus and phalanges.

**Figure 1:**
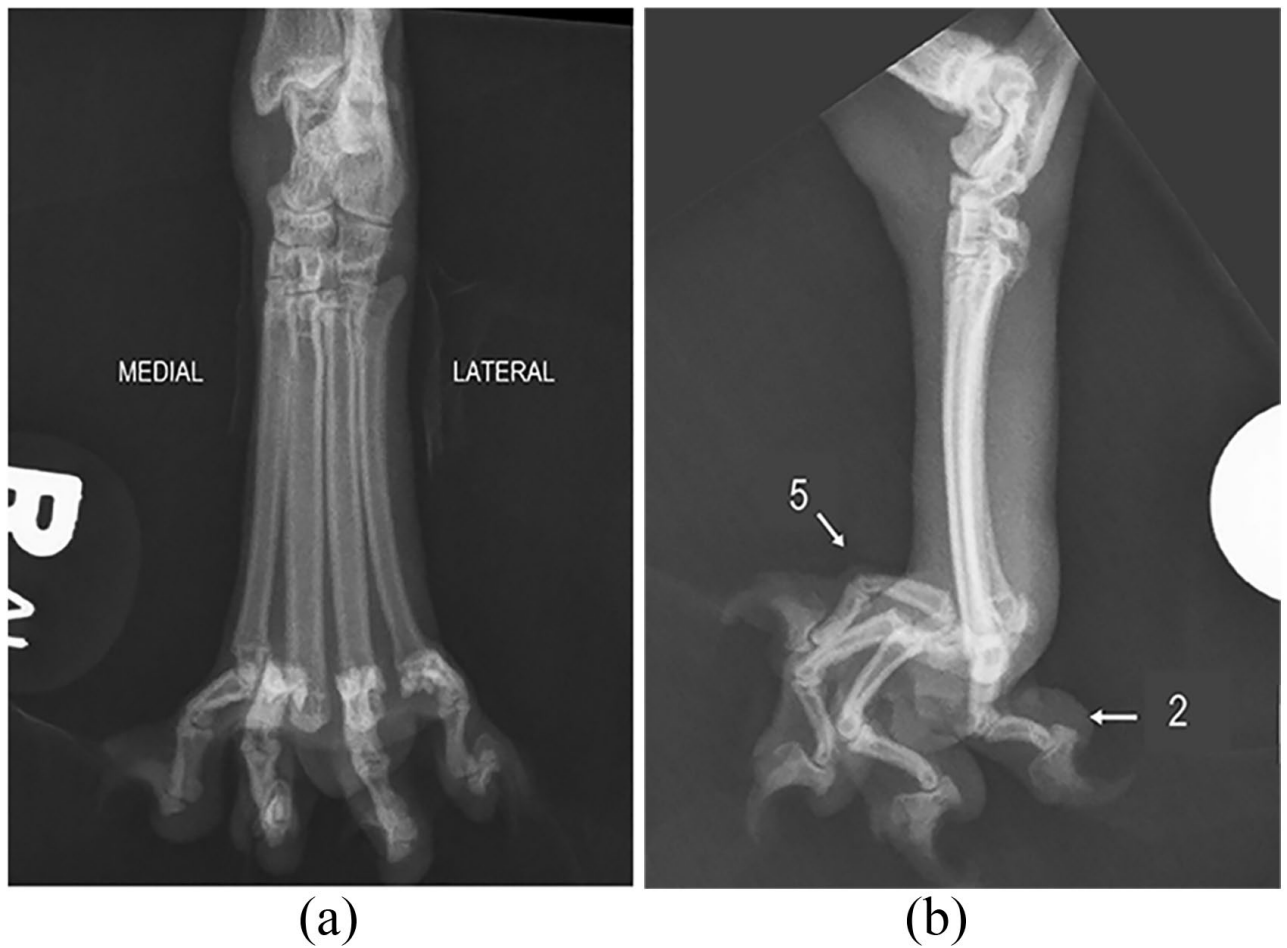
(a) Craniocaudal and (b) lateral radiographic images of the pes of the right pelvic limb. Dorsoproximal metatarsophalangeal luxation of digits III–V and lateral rotation of digit II are apparent with moderate associated soft tissue swelling

Radiographs of the right pes revealed dorsoproximal metatarsophalangeal joint luxations of the third, fourth and fifth digits, with moderate associated soft tissue swelling extending proximally to the tarsocrural joint (Figure 1). There was lateral rotation of the second digit relative to the head of the second metatarsal bone. Closed manual reduction of the metatarsophalangeal luxations was attempted under sedation but was unsuccessful.

**Figure 2:**
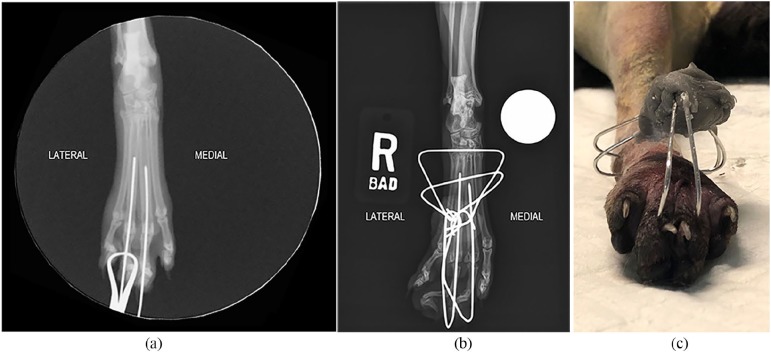
(a) Intraoperative craniocaudal fluoroscopic image of the pes of the right pelvic limb. The third, fourth and fifth metatarsophalangeal joints have been reduced. Digits III and IV are each stabilized with a transarticular and intramedullary Kirschner wire. (b) Postoperative craniocaudal radiographic image. Two transverse Kirschner wires have been placed and all four wires were bent to converge over the dorsal aspect of the pes. (c) Photographic image of the pes. A sphere of polymethylmethacrylate has been applied, which envelops the ends of all four Kirschner wires. Mild internal rotation of digits III and IV can also be observed

Open reduction and stabilization were performed under general anesthesia the following day. The cat was positioned in dorsal recumbency with the right pelvic limb extended caudally. A 2 cm dorsal incision was made, extending proximally from the metatarsophalangeal joint of the third digit. The digital extensor tendon was separated from the underlying bones and joint capsule using blunt dissection. Hohmann retractors were placed medial and lateral to the distal aspect of the third metatarsal bone to expose the metatarsophalangeal joint space. A Backhaus towel clamp was placed on the distal phalanx to traction the digit as a Freer elevator was used to successfully lever the proximal phalanx into reduction. The articulation was re-luxated and a pilot hole was made centrally in the articular surface of the head of the third metatarsal bone using a 1 mm Kirschner wire. The pilot hole completely penetrated the subchondral bone and appropriate positioning of the Kirschner wire in the medullary canal of the distal metatarsal bone was confirmed by fluoroscopy. The wire was removed and normograded from the proximal articular surface of the proximal phalanx, penetrating the dorsal cortex of the body of the phalanx. The wire was driven out of the skin overlying the proximal phalanx, until only 5 mm of wire was protruding proximal to the articular surface of the phalanx. The protruding segment of wire was inserted into the pilot hole and advanced into the medullary canal of the metatarsal bone. A second 2 cm dorsal incision was then made over the fourth metatarsophalangeal joint. Once the luxation was exposed, a Backhaus towel clamp was placed on the fourth distal phalanx and manual reduction was attempted but could not be obtained. The medial collateral ligament was transected in order to allow for reduction of the joint. A 1 mm Kirschner wire was then placed in a similar fashion as previously described, except that the proximal tip of the wire was trimmed before the wire was seated in the fourth metatarsal bone. Accurate positioning of the Kirschner wires was confirmed via fluoroscopy (Figure 2). The second and fifth metatarsophalangeal joints were then tractioned into reduction and were palpably stable. The surgical approaches were closed routinely in two layers.

A 1 mm Kirschner wire was percutaneously placed transversely through the base of metatarsal bones II–V. Radiographs were obtained and the transverse Kirschner wire was noted to impinge on the tarsometatarsal joints (Figure 2). This wire was not removed and a second Kirschner wire was placed transversely through the proximal third of the metatarsal bones II–V. Two pairs of heavy needle drivers were used to bend the protruding portion of the Kirschner wires to converge over the dorsal aspect of the pes. The transarticular wires were bent as close as possible to the proximal phalanx, while the transverse pins were bent 5–10 mm from the skin surface laterally or medially. A sphere of polymethylmethacrylate (Technovit 6 Treatment Kit; Jorgensen Laboratories) was applied, enveloping the ends of the Kirschner wires, and was allowed to polymerize. The skin surface was protected during application of the acrylic to prevent hyperthermic injury. A centimeter of clearance was maintained between the skin surface and the polymethylmethacrylate to allow for postoperative swelling (Figure 2).

The cat was willing to ambulate and bear weight on the right pelvic limb the morning following surgery, although there was marked swelling of the distal right pes, particularly digits III and IV. The cat was hospitalized for 48 h postoperatively, during which time the majority of swelling abated. The cat was discharged and the owner given instructions to administer meloxicam (0.1 mg/kg PO q24h) and buprenorphine (0.01 mg/kg sublingually q8–12h) for 3–5 days. Strict cage confinement was recommended and the owners were instructed to clean the wire–skin interfaces daily with dilute chlorhexidine solution followed by application of triple antibiotic ointment.

The cat was ambulating well when evaluated 1 week after surgery, but had a persistent weight bearing right pelvic limb lameness. The swelling had resolved, but there was visible internal rotation of the fourth digit. By 3 weeks, the cat was consistently bearing substantial weight on the right pelvic limb. The two wires stabilizing the luxations had minor wire tract inflammation.^1^ Radiographs revealed a moderate increase of the right third and fourth metatarsophalangeal joint spaces, and a mild amount of smooth periosteal proliferation along the plantar aspect of the proximal- to mid-diaphysis of the fifth metatarsal bone adjacent to the distal transverse Kirschner wire. Internal rotation of the third and fourth distal metatarsal bones and digits was also noted. All implants were removed and subsequent radiographs obtained, which showed markedly improved rotational alignment of the digits. The pes was placed in a plantar metasplint that extended to the proximal aspect of the calcaneus fashioned from thermoplastic material (Vet-lite Thermoplastic Casting Materials; Veterinary Specialty Products) for an additional week. At splint removal, the wire tract inflammation had resolved and the cat had a mild weight bearing lameness.

**Figure 3:**
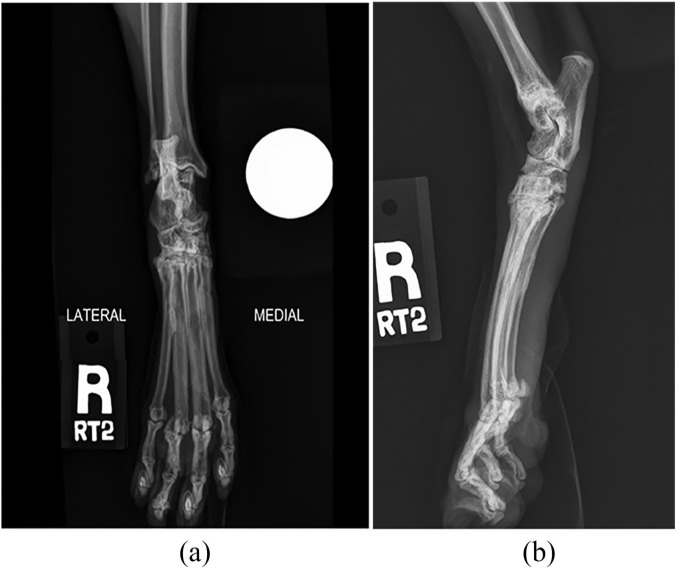
(a) Craniocaudal and (b) lateral radiographic images of the right pes obtained 6 months after surgery. There is moderate lateral displacement of the fourth metatarsophalangeal joint and both associated sesamoid bones, mild osteoarthrosis of the third and fourth metatarsophalangeal joints, and moderate osteoarthrosis of the tarsometatarsal and intertarsal joints

The cat was re-evaluated 6 months after surgery, at which time no lameness was observed. The third and fourth metatarsophalangeal joints were immobile on palpation, while the tibiotarsal and interphalangeal joints had maintained a normal range of motion. Pain was not elicited on manipulation of the pes or tarsus. Radiographs revealed moderate lateral displacement of the fourth digit and associated sesamoid bones relative to the fourth metatarsal bone (Figure 3). There was mild metatarsophalangeal osteoarthrosis of the third and fourth digits. In addition, there was mild distal intertarsal and moderate tarsometatarsal osteoarthrosis.

## Discussion

Metacarpophalangeal and metatarsophalangeal joint luxations occur infrequently in cats.^2^ Closed reduction should be attempted and may be successful in acute injuries.^2^ Open surgical stabilization of these injuries has been recommended if closed reduction is not attainable or if the luxation reccurs.^2^ Previously described surgical techniques include placement of ligament prostheses, as well as imbrication of the disrupted joint capsule and collateral ligament repair.^2^

The surgical technique used in this cat was a modification of the procedure described by Fitzpatrick et al for the stabilization of metacarpal and metatarsal fractures.^3^ The modifications included: (1) that the Kirschner wires were placed as transarticular implants rather than just intramedullary ones that penetrated only the distal articular surface of the fractured bone being stabilized; and (2) that the Kirschner wires were placed from the articular surface in normograde fashion rather than in retrograde fashion. The tip of the wire placed in metatarsal IV was blunted so the wire would not inadvertently penetrate the cortex, and we would advise blunting all wire tips in future cases.

The first, proximal transverse wire was inadvertently placed too proximal and impinged on the tarsometatarsal joints. We elected to leave the wire in place as we assumed the iatrogenic articular trauma was irreversible and unlikely to progress over the 3 weeks that the fixator would be maintained. Radiographic evaluation at 6 months postoperatively revealed moderate tarsometatarsal joint osteoarthrosis that was attributed to the placement of the proximal transverse wire. A second transverse Kirschner wire was placed distal to the first to decrease the mechanical demands on the initial wire and because this additional second wire might be needed should the initial wire require premature removal.

There was considerable postoperative swelling of the third and fourth digits which regressed within 48 h of surgery. Interestingly, swelling was reported in 50% of cats, but none of the dogs in Fitzpatrick et al’s study, suggesting that anatomic species-specific variations unique to cats may be a causative factor.^3^

The cat reported here also had distortion of the paw, specifically internal rotation of the central two digits in which reduction was maintained by the transarticular Kirschner wires. Paw distortion was reported in 10% of cats in Fitzpatrick et al’s study and was suspected to be secondary to impingement of the proximal phalanx by the wires.^3^ In the cat reported here, the transarticular wires penetrated the dorsal surface of the body of the proximal phalanx. We secured the protruding segment of wire in the jaws of a needle driver and bent the wire back on itself with a second needle holder. This resulted in nearly a 1 cm segment of wire protruding from the proximal phalanx, which likely impinged on the middle phalanx causing the internal rotation of both digits observed postoperatively. We would suggest withdrawing the Kirschner wires by 1 cm before bending the wire in future cases. The wire could then be re-advanced proximally with a pair of needle holders until the bend in the wire was in contact with the dorsal cortex of the proximal phalanx, which might alleviate impingement of the middle phalanx and prevent distortion of the secured digits.

Minor wire tract inflammation was observed 3 weeks postoperatively.^1^ Wire tract discharge was also commonly reported in cats (21%) in Fitzpatrick et al’s study,^3^ and we suspect that both the wire impingement and inflammation likely contributed to the cat’s lameness observed while the fixator was in place. Deformity of the digits and wire tract inflammation both spontaneously resolved after fixator removal, which coincided with a substantial improvement in weight bearing. These observations are congruent with those reported by Fitzpatrick et al, as cats were noted to be mildly to moderately lame while the fixator was in place, but only 2/13 cats were intermittently lame at the time of long-term evaluation.^3^ A metasplint was placed and maintained for 7 days after fixator removal in the cat reported here. External coaptation was not used following fixator removal in any cats in Fitzpatrick et al’s study^3^ and may therefore not have been necessary in the cat reported here.

There was persistent lateral subluxation of the fourth metatarsophalangeal joint on the radiographs obtained at the time of the final mid-term evaluation.^4^ The fourth digit also had substantial internal rotation while the fixator was in place compared to the third digit. During surgery we were forced to incise the medial metatarsophalangeal collateral ligament to obtain reduction and we ascribe subluxation and increased internal rotation to transecting this ligament. Alternatively, subluxation and internal rotation could have been caused by impingement of the Kirschner wire stabilizing this digit on the middle phalanx.

The development of degenerative osteoarthrosis was not surprising, since Fitzpatrick et al reported this to be the most common complication of this stabilization technique, affecting 54% of cats in their study.^3^ Even though the cat in the current report had no appreciable lameness 6 months after surgery, the more long-term effects of these degenerative changes are currently unknown.

## Conclusions

The combined transarticular and external skeletal fixation used in the cat reported here allowed for successful stabilization of the metatarsophalangeal joints and resolution of lameness within 6 weeks of surgery, with only mild associated complications. Application of the construct was considered simple and cost-effective. Possible advantages of this technique over imbrication and ligament prosthesis include the fixation obviates the need for external coaptation, would provide unobstructed access for wound management if needed and that the implants can be readily removed when no longer needed.
